# Infectious eccrine hidradenitis caused by *Mycobacterium chelonae* in a patient with sarcoidosis

**DOI:** 10.1016/j.jdcr.2022.10.015

**Published:** 2022-10-31

**Authors:** Mary McDaniel, H. Nicholas Shamma, Jaclyn Wetli

**Affiliations:** a Department of Dermatology, Wright State University, Fairborn, Ohio; b American Dermatopathology Laboratory, Centerville, Ohio; c CAPS Dermatology, Upper Arlington, Ohio

**Keywords:** atypical mycobacterium, infectious eccrine hidradenitis, immunosuppression, *Mycobacterium chelonae*, *M. chelonae*, neutrophilic eccrine hidradenitis, sarcoidosis, IV, intravenous

## Introduction

*Mycobacterium chelonae* (*M. chelonae*) is a widely prevalent nontuberculous mycobacterium.^1^ Infections from *M. chelonae* are typically limited to the skin, though the disseminated disease can be seen in immunosuppressed individuals.^2^ Clinical features of cutaneous *M. chelonae* are variable from papules and plaques to abscesses and cellulitis.^1^^,^^2^ Here we describe an unusual case of a cutaneous *M. chelonae* infection in a patient with sarcoidosis. To the best of our knowledge, there is only one other report showing the histopathologic features of neutrophilic eccrine hidradenitis caused by *M. chelonae*.

## Case report

A 69-year-old female presented to the outpatient dermatology office with a 5-month history of a nonhealing lesion on her left arm. She was hospitalized with COVID-19 in July 2021 during which an intravenous (IV) line was placed at the site of the lesion. The IV site was also confirmed by her primary care physician who had seen her shortly following hospital discharge. Her medical history was significant for pulmonary sarcoidosis treated with prednisone 20 mg every other day and methotrexate 15 mg weekly since 2013. That September, infliximab was added to her medication regimen as there was concern that her pulmonary sarcoidosis was progressing, which was demonstrated by worsening pulmonary function testing. The patient initially noticed the lesion 1 month after her first infliximab infusion and reported that it had since continued to grow steadily.

**Fig 1:**
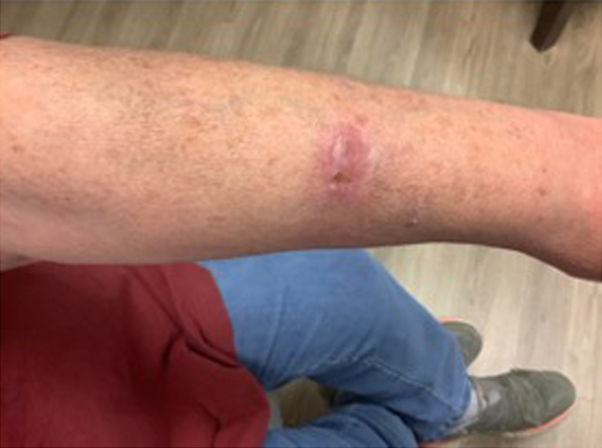
Ill-defined, erythematous, crusted keloidal plaque on the left side of the dorsal aspect of the forearm.

On physical exam, there was an ill-defined, crusted, erythematous, keloidal plaque on the left dorsal forearm. Additionally, there were tender, subcutaneous nodules with no overlying epidermal change proximally on the left portion of the volar aspect of the arm in a sporotrichoid pattern (Fig. 1).

**Fig 2:**
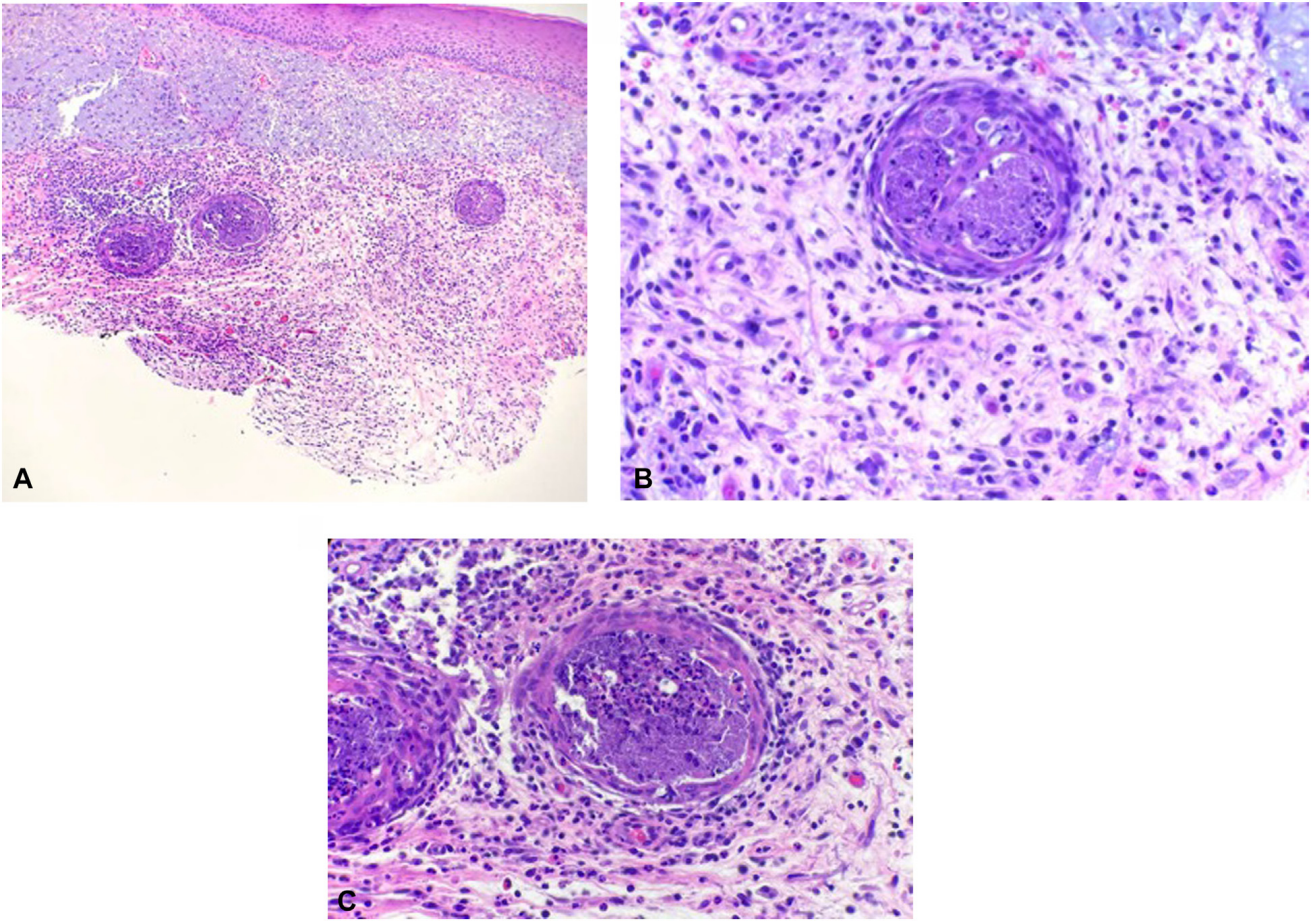
**A,** Mixed inflammatory infiltrate within the dermis and suppurative inflammation centered within and around the eccrine glands. **B** and **C,** suppurative inflammation of the eccrine gland with surrounding histiocytes, plasma cells, and eosinophils.

A shave biopsy of the forearm lesion revealed suppurative and granulomatous dermatitis with eosinophils and plasma cells focally involving the eccrine glands. Periodic acid–Schiff and acid-fast bacillus stains were negative. No foreign body material was seen with polarized light. Given the unusual pathology, tissue cultures were obtained, and grew *M. chelonae*. The patient was started on clarithromycin 500 mg twice per day, referred to infectious disease, and infliximab and methotrexate were held. Disseminated infection was not found on further investigation. The patient showed complete clearance of all skin lesions following clarithromycin monotherapy for 3 months. Her pulmonary function improved following the discontinuation of methotrexate and infliximab. She is now stable on mycophenolate mofetil which was added by her pulmonologist once she had completed the course of clarithromycin (Fig. 2).

## Discussion

Given the range of presentations for atypical mycobacterium infections, clinical suspicion combined with histopathologic examination and tissue culture is essential to confirm the diagnosis. Infection with *M. chelonae* typically results from direct inoculation.^3^ Other cases have been reported demonstrating being infected with *M. chelonae* following cosmetic injections, other medical procedures, tattoos, and acupuncture.^1^^,^^4^ In our case, the infection likely resulted when *M. chelonae* was introduced into the skin during the insertion of an IV line. Our patient was at higher risk for contracting an infection during a routine IV line placement as a result of her suppressed immune system.

Histopathologic features of atypical mycobacterium infections are heterogeneous and dependent on the immune status of the individual.^5^ This case is unique in that the significant pathologic findings were concentrated around eccrine glands, emulating neutrophilic eccrine hidradenitis with subtle granulomatous dermatitis. Diminished granuloma formation among immunosuppressed patients has been reported in cases of atypical mycobacterium infections, though we postulate that the lack of an expected diffuse infiltrate in our patient was related to the medications for her sarcoidosis, which blunted a typical inflammatory response.^5^

Neutrophilic eccrine hidradenitis is classically associated with hematologic malignancies and chemotherapy; however, it is less commonly seen with infectious etiologies and, in such cases, has been termed as infectious eccrine hidradenitis.^6^ One confounding source, in this case, is the patient’s history of taking methotrexate, which is a reported cause of neutrophilic eccrine hidradenitis. However, given that the patient had been stable on methotrexate for years with no prior issues, this was unlikely to be the source of the pathologic findings.^7^ This case represents one of the few reported cases of infectious eccrine hidradenitis caused by *M. chelonae* and demonstrates the importance of considering an infectious etiology when findings consistent with neutrophilic eccrine hidradenitis are seen on the biopsy in an immunosuppressed patient.

## Conflicts of interest

None disclosed.

## References

[1] Akram S.M., Rathish B., Saleh D. (2022).

[2] Park M.A., Gaghan L.J., Googe P.B., Klein K.R., Mervak J.E. (2021). Disseminated cutaneous *Mycobacterium chelonae* infection as a presenting sign of ectopic adrenocorticotropic hormone syndrome. JAAD Case Rep.

[3] Popli U., Rawleiy E., Yesudian P.D. (2020). Tender cutaneous nodules in a patient with cardiac sarcoidosis. Clin Exp Dermatol.

[4] McCallum C., Johnston B. (2016). Mycobacterium chelonae bacteremia in a patient taking infliximab. CMAJ.

[5] Sardiña L.A., Kaw U., Jour G. (2020). Diagnosis of Mycobacterium abscessus/chelonae complex cutaneous infection: correlation of tissue culture and skin biopsy. J Cutan Pathol.

[6] Bassas-Vila J., Fernández-Figueras M.T., Romaní J., Ferrándiz C. (2014). Infectious eccrine hidradenitis: a report of 3 cases and a review of the literature. Acta Dermosifiliogr.

[7] Mills L., Steinmetx-Rodriguez C., Folkes A., Shecter R. (2017). Neutrophilic eccrine hidradenitis: An unusual case and a review of the literature. JAOCD.

